# Quinine against Obstinate Hiccup

**Published:** 1844-11

**Authors:** 


*Quinine against obstinate Hiccup.*—Among the many anomalous, and not unfrequently very obscure, results of miasmatic influence, are various affections of the digestive organs. Of these, Hiccup is a most troublesome, and, occasionally, a very intractable one. In some cases, this gastric disorder has been observed to exhibit a distinctly intermittent character, and to be associated with the existence of other phenomena which are usually attributed to the operation of the same morbific cause. M. Mendiere has recently published, in the pages of the Revue Medicale, several cases of this sort, in which the distressing symptom of Hiccup was promptly and decisively cured by the free use of quinine, after it had resisted every other mode of treatment. He has used the same remedy with good results in many cases of severe Cardialgia.

(The addition of a few drops of tinct. opii to the quinine will greatly enhance its efficiency in such cases. What an admirable remedy opium is in almost all the Neuroses, provided the secretions are healty at the time! The difficulty lies in knowing how to time its administration, and regulate its dose. We have usually found the liquor opii sedativus the form in which to exhibit it: this may be given in a bitter infusion, to which ammonia may generally be added with advantage at the same time.)—*Ibid.*

